# Clinical DRUJ instability does not influence the long-term functional outcome of conservatively treated distal radius fractures

**DOI:** 10.1007/s00068-015-0627-4

**Published:** 2016-01-29

**Authors:** M. M. E. Wijffels, P. Krijnen, I. B. Schipper

**Affiliations:** Department of Surgery-Traumatology, Leiden University Medical Center, P.O. Box 9600, 2300 RC Leiden, The Netherlands

**Keywords:** Distal radioulnar joint, Instability, Distal radius fracture, Wrist

## Abstract

**Objective:**

The presence of distal radioulnar joint (DRUJ) instability remains often unnoticed initially, but may worsen functional outcome of distal radius fractures (DRF). The aim of this study was to evaluate the influence of concurring clinical DRUJ instability on the outcome of conservatively treated DRF.

**Methods:**

In a retrospective cohort study, all unilateral, conservatively treated DRF patients were invited for physical examination, CT scan of both wrists and filling out questionnaires. Static and dynamic DRUJ instability were clinically tested.

**Results:**

Forty-nine DRF patients with a mean follow-up of 4.2 years (SD 0.5) were assessed. Seventeen patients tested positive for DRUJ instability. No differences were found in baseline characteristics between the DRUJ stable and unstable group. Apart from wrist flexion, no statistical significant differences in outcome was found between patients with and without DRUJ instability.

**Conclusion:**

The presence of clinical DRUJ instability does not seem to affect functional outcome of conservatively treated distal radius fractures at long-term follow-up.

## Introduction

Distal radius fractures (DRF) comprise one in six fractures that present to the emergency department [1–3]. The treatment decision between surgical or conservative fracture management is based on several factors including the type and complexity of the fracture, bone quality, the surgeon’s experience and patient characteristics [4]. The majority (50–80 %) of DRF is still treated conservatively, although there is an increasing tendency towards surgical treatment [5, 6].

One of the complicating factors with a potentially negative influence on the outcome of DRF treatment is concurring distal radial ulnar joint (DRUJ) instability. The incidence of DRUJ instability after DRF varies from 0 to 35 % at 1 year after a distal radius fracture [7–10] . Lindau found that DRUJ instability was independently associated with worse outcome after DRF, showing significantly more pain and lower wrist scores for DRUJ unstable patients [7, 8]. These findings resulted from DRF patients after a variety of treatment modalities, amongst which surgery. In this study, the surgery itself may have influenced the outcome [7, 8]. Currently, it is unknown how the outcome of non-operatively treated DRF is influenced by coexisting DRUJ instability. The aim of this study therefore was to assess the influence of clinical DRUJ instability on the long-term outcome of conservatively treated DRFs.

## Patients and methods

All adults treated conservatively for a DRF between May 2008 and February 2010 in the Leiden University Medical Center were eligible for inclusion in this study. Patients were excluded if they were (1) unwilling or unable to provide informed consent or had (2) systemic diseases such as rheumatoid arthritis and SLE, or had (3) contralateral wrist injury. All eligible patients received an invitational letter for a study visit according to the protocol, as approved by the institutional medical ethics review board.

### Visit

During the research visit DRUJ instability of both the injured and non-injured wrist was tested by an experienced trauma surgeon not involved in the initial treatment and unaware of the injured side. Static instability was tested using the stress test [8, 11, 12]. The test was considered positive if there was more anterioposterior movement, in any direction, of the ulna relative to the stabilized radius in the injured wrist compared to the uninjured wrist. Dynamic instability was tested using the clunk test [13]. The test was considered positive if a “clunk” was palpable for either the patient or examiner, during pronosupination of the lower arm with the wrist held between the examinator’s thumb and index finger.

Further physical examination of both wrists consisted of measurement of range of motion and grip- and pinch strength for both the injured and non-injured wrist. Range of motion was measured using a goniometer. Grip and pinch strength were based on the mean value of three successive measurements using a Jamar^®^ Hydraulic Hand Dynamometer and a Jamar^®^ Hydraulic Pinch Gauge, respectively. Maximum range of motion of the injured wrist was expressed in degrees and as a percentage of the non-injured wrist.

### Radiological evaluation

Conventional trauma radiographs in lateral and anterioposterior view were evaluated by one observer (MW) to determine the fracture classification according to the Comprehensive Classification of Fractures [14]. CT scans of both wrists were obtained and evaluated for radiocarpal arthritic changes according to the classification of Knirk and Jupiter and for union of the ulnar styloid, if a fracture was previously present [15]. The CT scans were made at 3-mm intervals using an Aquilion One (Toshiba Medical Systems). Axial sections of the wrist were obtained with the arm outstretched position above the head in prone position to minimize the exposure to radiation.

### Patient reported outcome

Participants scored pain on a visual analog scale five times for the posttraumatic wrist, indicating perceived pain (1) in rest, (2) with 10 kg loadbearing with the elbow extended and (3) with 10 kg loadbearing with the elbow 90° flexed, (4) during pronosupination without loadbearing and (5) during pronosupination with 10 kg of loadbearing. Furthermore, the Disabilities of the Arm, Shoulder and Hand (DASH) score, Modified Mayo Wrist Score (MMWS) and Gartland and Werley score were filled out by the participants [16–18].

### Statistical analysis

Baseline characteristics and outcome scores of the clinically DRUJ stable and unstable groups were compared. The static DRUJ unstable group was compared to the stable group, as was the dynamic DRUJ unstable group. Continuous data were compared between groups by the independent samples *t* test, categorical data by the Pearson’s Chi-square test. Analyses were performed using SPSS version 20 (SPSS Inc, Chicago IL). *p* values below 0.05 were considered statistically significant.

## Results

In total, 156 patients met the inclusion criteria and were sent an invitational letter for a study visit. Thirty-four patients were lost to follow-up and 73 were unwilling to participate. The resulting 49 participants provided written informed consent before examination. No differences were found in age, gender or fracture classification comparing participants with no participants (data not shown).

The study group consisted of 10 men and 39 women, both distributed without a significant difference over the DRUJ stable and unstable group Mean age was 60.8 years (SD 16.2). Mean follow-up after trauma was 4.2 years (SD 0.5). Seventeen of the 49 patients tested positive for DRUJ instability. The groups with and without DRUJ instability were similar with respect to age, gender, dominant hand injured, fracture characteristics and degree of radiocarpal arthritis. No arthritic changes of the DRUJ were found. In the DRU stable patients 13 patients had an accompanying ulnar styloid process (PSU) of which 7 united. In the DRU unstable patients 11 patients had an accompanying ulnar styloid process (PSU) of which 4 united. No significant differences were found in PSU-union between groups (Table 1).

**Table 1:** Baseline characteristics of patients with static distal radioulnar joint instability compared to patients with a stable distal radioulnar joint

	DRUJ stable (*n* = 32)	DRUJ instability (*n* = 17)	*p* value
Age (years) [SD]	61.5 [14.6]	59.5 [19.3]	.686
Follow-up (years) [SD]	4.2 [0.5]	4.1 [0.5]	.767
Gender			.07
Male	9	1	
Female	23	16	
AO classification (*n*)			.448
A	13	10	
B	4	1	
C	15	6	
Dominant hand injured (*n*)			.426
Yes	15	10	
No	17	7	
PSU-union (*n*)			.185
Yes	7	4	
No	6	7	
No PSU fracture	19	6	
Radio carpal arthritis (*n*)			.138
0	1	3	
1	14	7	
2	13	3	
3	4	4	

0, none; 1, slight joint-space narrowing; 2, marked joint-space narrowing, osteophyte formation; 3, bone-on-bone, osteophyte formation, cyst formation

### Range of motion and strength

In patients with an unstable DRUJ flexion and extension averaged 81° (range 58°–102°) and 89° (range 58°–110°), respectively. Radial deviation averaged 25° (range 5°–40°) and ulnar deviation averaged 40° (range 25°–58°). The average pronation and supination measured 84° (range 60°–100°) and 89° (range 75°–110°), respectively. In patients with a stable DRUJ of the injured wrist flexion averaged 68° (range 35°–92°) and extension 85° (range 55°–105°). Radial deviation averaged 24° (range 6°–40°) and ulnar deviation averaged 40° (range 21°–65°). The average pronation and supination measured 87° (range 68°–109°) and 92° (range 60°–118°), respectively. Function expressed as percentage of the uninjured arm is shown in Table 2. Flexion was found to be statistical significantly better in the DRUJ unstable group. Further results did not differ between patients with and without DRUJ instability.

**Table 2:** Functional outcome parameters of patients with stable or unstable distal radioulnar joint

	DRUJ stable (*n* = 32)	DRUJ unstable (*n* = 17)	*p* value
Flexion in degrees, mean (range)	68 (35–92)	81 (58–102)	
% of non-injured wrist	0.93 (0.13)	1.01 (0.15)	.04
Extension in degrees, mean (range)	85 (55–105)	89 (58–110)	
% of non-injured wrist	1.0 (0.11)	1.00 (0.10)	.88
Pronation in degrees, mean (range)	87 (68–109)	84 (60–100)	
% of non-injured wrist	0.98 (0.09)	0.98 (0.09)	.95
Supination in degrees, mean (range)	92 (60–118)	89 (75–110)	
% of non-injured wrist	1.02 (0.15)	0.98 (0.14)	.28
Radial deviation in degrees, mean (range)	24 (6–40)	25 (5–40)	
% of non-injured wrist	1.1 (0.36)	1.10 (0.40)	.89
Ulnar deviation in degrees, mean (range)	40 (21–65)	40 (25–58)	
% of non-injured wrist	1.0 (0.61)	0.97 (0.20)	.68
Grip strength, % of non-injured wrist	0.93 (0.13)	0.98 (0.12)	.25
Pinch strength, % of non-injured wrist	0.95 (0.22)	1.01 (0.22)	.34
DASH	7.9 (16.3)	10.05 (9.72)	.63
MMWS	87.03 (12.9)	87.06 (10.47)	.99
Gartland and Werly	4.2 (3.66)	4.9 (3.47)	.49
Pain injured wrist in rest	4.7 (16.7)	5.3 (15.05)	.90
Pain injured wrist, carrying 10 kg, with elbow extended	9.3 (18.7)	11.8 (23.78)	.69
Pain injured wrist, carrying 10 kg, with elbow flexed	7.7 (19.6)	10.6 (20.5)	.63
Pain injured wrist during pronosupination without load	3 (11.4)	0 (0)	.29
Pain injured wrist during pronosupination with 10 kg loadbearing	8.9 (21.1)	11.2 (22.05)	.72

Results are expressed as mean (SD) unless indicated otherwise

In patients with an unstable DRUJ the grip and pinch strength averaged 98 % (range 59–115 %) and 101 % (range 51–133 %), respectively, indexing the uninjured for the injured wrist. In patients with a stable DRUJ, the grip and pinch strength averaged 93 % (range 63–132 %) and 95 % (range 50–161 %), respectively, indexing the uninjured for the injured wrist. No differences in strength were found between the groups (Table 2).

### Patient reported outcome

Ten of the DRUJ unstable patients revealed only static DRUJ instability, of which two suffered from wrist pain in rest and seven patients showed both static and dynamic instability, of which one suffered from wrist pain in rest. Two patients suffered from pain prior to the trauma, in the injured wrist.

In the DRUJ unstable group, three patients indicated pain in the injured wrist in rest with a mean of 5.3 (range 0–50). The mean outcome for pain, with 10 kg loadbearing with the elbow extended, with 10 kg loadbearing with the elbow 90° flexed, during pronosupination without loadbearing and during pronosupination with 10 kg of loadbearing on the VAS was 11.8 (range 0–70), 10.6 (range 0–60), 0.0 (range 0–0) and 11.2 (range 0–70), respectively. Mean DASH score was 10 (range 0–28). One patient did not fulfill the DASH. The MMWS was excellent or good in 16 and poor in one patient. The Gartland and Werley ratings were excellent in 5 patients, good outcome in 9 patients and fair in 3 patients.

In the DRUJ stable group, three patients indicated pain in the injured wrist in rest with a mean of 4.7 (range 0–80). Average outcome for pain with 10 kg loadbearing with the elbow extended, with 10 kg loadbearing with the elbow 90° flexed, during pronosupination without loadbearing and during pronosupination with 10 kg of loadbearing on the VAS was 9.3 (range 0–60), 7.7 (range 0–90), 3.0 (range 0–60) and 8.9 (range 0–80), respectively. Mean DASH score was 7.9 (range 0–68). The MMWS ratings were excellent or good in 25 patients, satisfactory in 5 and poor in 2 patients. The Gartland and Werley ratings were excellent and good outcome in 25 patients and satisfactory or poor in 7 patients.

There were no significant differences between DRUJ stable and instable groups in terms patient reported outcomes (Table 2). Comparing patients with static or dynamic DRUJ instability separately to stable DRUJ patients, no statistical significant differences were found (data not shown).

## Discussion

Concurring DRUJ instability is assumed to be an independent factor for poor functional outcome in conservatively and operatively treated DRF [8]. This study analyzed the relation between DRUJ instability and functional outcome in a group of conservatively treated DRF patients and found that patients with clinical DRUJ instability, after consolidation of the DRF, had a long-term functional outcome similar to that of patients after DRF without DRUJ instability with a significantly better wrist flexion. To our knowledge, no previous studies focussed on conservatively treated patients solely.

### Function

In the present study, only a significant better flexion of the wrist was found in the DRUJ unstable patients. No further differences in function or strength were found, comparing patients with a stable DRUJ to patients with an unstable DRUJ. Since flexion and extension of the wrist primarily concern the radiocarpal joint and not so much the DRUJ, finding a difference was not anticipated for this motion. The greater range of flexion in the DRUJ unstable group may be spurious given the relatively small number of patients and the absence of other plausible explanations. However, the expected difference in pronosupination, a movement mainly involving the DRUJ, was not found. A reason for this may be the influence of muscle dependent stabilizers of the DRUJ, such as the extensor carpi ulnaris tendon, the pronator quadratus muscle and the radioulnar interosseous membrane [19–21]. During pronosupination the muscles tighten resulting in a more stable DRUJ, independent of ligamentous stabilization. These muscle-tension dependent stabilizers may also be the reason for absence of difference in grip- and pinch strength; a finding corresponding with the results of Lindau et al. [22]. Since muscle relaxation is warranted during the clinical tests, the DRUJ is no longer supported resulting in a positive testing result.

### Ulnar styloid

Distal radial ulnar joint instability results from malfunctioning of joint stabilizing structures. These stabilizers are, among others, ligamentous and therefore invisible on conventional radiographs and computed tomography (CT) without contrast. Secondary signs may be visualized using these radiological techniques. Since the triangular fibrocartilage complex (TFCC) attaches to the ulnar styloid process (USP), non-union of the USP may indicate loss of TFCC as a stabilizing factor. Static and dynamic DRUJ instability was found in 33 and 17 %, respectively, of the patients in the current study. No difference was found in the number of ulnar styloid non-unions in DRUJ unstable patients compared to DRUJ stable patients. This implies that the TFCC is not the only stabilizing structure and that non-union of the ulnar styloid has no influence on DRUJ stability, which corresponds with literature [9, 10, 23–26].

### Pain

According to Stoffelen et al., DRUJ instability may cause ongoing wrist pain [27]. Lindau et al. found no statistical difference in pain comparing lax DRUJ patients with DRUJ stable patients [22]. Similarly, in this study we found no statistically significant difference in pain between patients with or without clinical DRUJ instability. The absent difference in pain scores may be due to the long follow-up of this (4 years) and Lindau’s study (6 years), compared to the follow-up of 1 year in the study of Stoffelen et al. Still these conclusions should be interpreted with caution since ongoing pain after distal radius fractures can be caused by several factors which are not all evaluated in this and other studies [22, 28–30].

Arthritic changes of the DRUJ after chronic instability have been described [31]. For this reason, it is remarkable that no arthritic changes in the DRUJ were found, despite symptomatic wrists in some patients. These results may be caused by the fact that symptoms were not attributed to a specific wrist region, e.g., the DRUJ region or follow-up as too short to develop DRUJ arthritic changes. Furthermore most distal radius fractures did not involve the DRUJ and may therefore not result in cartilage damage, a finding that may be seen in a posttraumatic incongruent DRUJ.

### Shortcomings

The shortcomings of this study include the small number of patients analyzed, due to the loss of follow-up of many patients and refusal to participate of many others. However, no differences in age or fracture type was found comparing participant with no participants so the participants may be assumed to be representative for the whole group although we were not informed about residual complaints in non-participants. Another study limitation is that, although widely used, the reliability and accuracy of the tests for determination of clinical DRUJ instability is uncertain. [8, 13, 27, 32] This may be due to the subjective qualification of “more laxity compared to the non-injured wrist” when using the stress test. Since the tests were done only once, by one investigator we were not able to establish inter- and intra-observer variation. In the absence of other clinical tests, the stress test and clunk test were used to mimic daily clinical practice and enable comparison of the results with published literature.

Furthermore, no information was available on the presence of DRUJ instability before the distal radius fracture. This aspect is hard to evaluate in any study since this pre-trauma information is generally lacking. Mikic [33] showed an increasing percentage of TFCC tears during lifespan, suggesting that degenerative DRUJ instability may have been present in some of the patients.

## Conclusion

Based on this study with some shortcomings, DRUJ instability does not seem to affect clinical outcome of conservatively treated distal radius fractures after long-term follow-up. Conservative treatment of secondarily diagnosed DRUJ instability after a DRF, seems to be a justified treatment.
